# Skin antiseptics in healthcare facilities: is a targeted approach necessary?

**DOI:** 10.1186/s12889-019-7507-5

**Published:** 2019-08-22

**Authors:** Timothy L. Wiemken

**Affiliations:** Center for Health Outcomes Research, Saint Louis University, 3545 Lafayette Ave, #411, St. Louis, MO 63108 USA

**Keywords:** Antisepsis, Bathing, Chlorhexidine, Surgical preparation, Healthcare-associated infection, Antimicrobial stewardship

## Abstract

**Background:**

Skin antisepsis occurs in every healthcare environment. From basic hand hygiene, to antiseptic bathing and pre surgical care with alcohol/chlorhexidine, use of antimicrobial agents to reduce the skin microflora has skyrocketed in the past several years. Although used in hopes of reducing the likelihood of infection in patients, many products have been identified as the source of infection in several outbreaks, sometimes due to the nonsterile nature of the many readily available antiseptics.

**Body:**

Intrinsic contamination of antiseptics during the manufacturing process is common. In fact, since the majority of these products are sold as nonsterile, they are allowed some level of microbial contamination based on the United States Pharmacopeia documents 61 and 62. Unfortunately, sometimes this contamination is with microorganisms resistant to the antiseptic and/or with those pathogenic to humans. In this scenario, healthcare-associated infections may occur, leaving the patient at higher risk of mortality and increasing costs of care substantially. Although antibiotic stewardship programs throughout the world suggest targeting use of antibiotics to limit resistance, few healthcare environments include other antimicrobial agents (such as antiseptics) in their programs.

**Conclusion:**

Due to the potential for contamination with pathogenic organisms and the increased likelihood of selecting for resistant organisms with widespread use of broad-spectrum agents with non-specific mechanisms of action, a discussion around including skin antiseptics in stewardship programs is necessary, particularly those labeled as nonsterile. At minimum, debating the pros and cons of targeting use of daily antiseptic bathing in hospitalized patients should occur. Through mindfully incorporating any antimicrobial agent, sterile or not, into our repertoire of anti-infectives, we can save patient lives, reduce infection, and save costs.

## Background

Skin antisepsis in healthcare has a long and storied history, from the intense skepticism that Semmelweis met after suggesting the importance of hand hygiene in obstetrics [1], to the current near standard-of-practice antiseptic bathing of hospitalized patients [2–14]. When it comes to skin antisepsis, much of the discussion in practice is centered around which active ingredient(s) to use, largely for pre-surgical care [15], as uses and contraindications vary by ingredient. Povidone-iodine, alcohol, chlorhexidine, colloidal silver, and many other available skin antiseptics and combinations of antiseptics each have unique pros and cons, which are well documented [15–21].

Although selection of a product is typically focused on these active ingredients, more recently a discussion has begun regarding the importance of understanding the preparation and manufacturing of the products themselves [16–18]. The majority of skin antiseptic products used in healthcare are marketed as nonsterile, meaning even after packaging, the product is allowed to have some bacterial contamination. Although skin antiseptics have antimicrobial properties by definition, they do not and cannot completely eliminate organisms on the skin. Further, many pathogens have reduced susceptibility, as well as intrinsic or acquired resistance to some of the active ingredients. These factors may have contributed to several outbreaks [21].

The purpose of this article is to begin the discussion of the importance of targeting use of skin antiseptic products in hospitalized patients to reduce the likelihood of infection, and also introduce the salient concepts surrounding infection, skin antisepsis, and contamination of antiseptic products.

## Main text

### Manufacturing skin antiseptics

The process of creating and packaging a skin antiseptic product for use in healthcare is complex. Generally, skin cleansers and antiseptics are sold as nonsterile products. Because of this, some microbial contamination is allowed. During preparation and packaging, the manufacturer must follow the United States Pharmacopeia documents 61 and 62 [22, 23], outlining the methods to enumerate and examine microbial contamination in their products. These documents provide specific testing approaches and evaluation of results to ensure that products are not contaminated with large amounts of what are termed ‘objectionable organisms’ - largely those capable of causing infection, particularly in an immunocompromised host.

### Contamination of skin antiseptics

Contamination of nonsterile skin antiseptic products may be labeled as extrinsic or intrinsic based on if the contamination occurred during the manipulation of the product after manufacturing, or during the manufacturing process, respectively. The major discussion recently is on intrinsic contamination during the manufacturing process. Although less common, intrinsic contamination has led to many outbreaks through use of non-sterile alcohol prep-pads [24], chlorhexidine [25–27], and other basic cleansers [28]. In fact, these issues can be traced back many decades [21]. Although likely to be under-reported due to the lack of adequate understanding and identification of the source of many outbreaks, the documentation of these outbreaks remains relatively rare.

### The potential for sterilization of skin antiseptics

As mentioned previously, intrinsic contamination occurs when the product is being made. Since skin antiseptics are largely water-based, every water-based manufacturer will have this issue unless the product can be or is sterilized after packaging. Biofilms containing microorganisms in water systems are difficult or impossible to eliminate, and therefore some organisms will inevitably contaminate the final product during the manufacturing process. The major solution to this may seem simple: sterilize the final product. Sterilizing the product after manufacturing but prior to release for sale is a simple and effective way to reduce the likelihood of infection on use of any product. Unfortunately, some of the ingredients may be inactivated by common processes used to sterilize, therefore most may have some level of microbial contamination and are released and labeled as nonsterile products. Only recently are manufacturers beginning to identify novel ways to sterilize products such as chlorhexidine, which were previously considered difficult to impossible of being sterilized after production [29].

### Infection related to skin antiseptics

In healthy individuals, a small amount of even an objectionable microorganism on the skin is unlikely to cause an issue. However, if that organism gains access to a sterile body site through a medical intervention or self-inoculation, infection is possible. In healthy individuals, the skin microflora may provide some colonization resistance to an organism applied to the skin [19]. This means that a healthy, robust normal flora on the skin reduces the chances that an objectionable organism placed in the skin is capable of maintaining colonization. However, the goal of skin antisepsis is to reduce the bioburden of all organisms on the skin [20]. If this occurs as planned, the normal skin flora is reduced, allowing for easier colonization access to the objectionable organism. Since this organism must be tolerant or resistant to the skin antiseptic having resided in the product, the patient now has a compromised skin flora and a new, objectionable organism on the skin selected to be resistant to the antiseptic. There is little research in this area with respect to the likelihood of infection in healthy individuals, but for those with compromised immunity, the risk is real and documented to such an extent that a review was conducted of all of the reports up to 2007 [21]. This begs the question as to if we should include antiseptics in our antimicrobial stewardship programs, as suggested by leaders in the field [30], or at minimum have a more informed discussion of the potential impact of providing targeted skin antisepsis with various products based on their likelihood of patient harm. After all, our failures to understand basic antimicrobial use in the past have led to our current antibiotic resistance issues [31]. Because of this, it is reasonable to believe that that overuse of antiseptics with broad, non-specific mechanisms of action and little understanding of the higher-level effects on the skin microbiota will put us in a similar predicament in the future.

### Targeting antisepsis

Targeted antisepsis could be accomplished in many different ways, and if under the authority of antimicrobial stewardship programs, could be monitored and enforced. Since the goal of antisepsis is to kill organisms, just like antibiotics, we should take a similar approach to ensure the right drug (active ingredient in the antiseptic), in the right dose, for the right duration, in the right patient should be accomplished. Targeted use is critical to ensure we reduce the likelihood of infection due to any amount of contamination of nonsterile skin antiseptics and to ensure we do not lose these valuable treatments to antimicrobial resistance.

This area is multifaceted, with little evidence available to argue outside of expert opinion. However, given the recent infections due to nonsterile skin antiseptics, it is clear that practitioners may be unaware that many of these products may be intrinsically contaminated with potentially harmful organisms. Applying these organisms to the skin may lead to infection, a risk that should be considered and closely monitored in a facility risk assessment if these products are used.

## Conclusions

Many interventions at various levels could be developed to limit the potential for infection due to nonsterile skin antiseptic use in healthcare. At the policy level, closer evaluation of processes and outcome measures by regulators may be critical for any product labeled as nonsterile and place on the skin of immunocompromised individuals. Requirement of sterility is another potential intervention, though with both of these issues, cost-effectiveness and cost-benefit calculations including a variety of outcomes from a variety of perspectives will be critical. Without this, a direct benefit to patients may not be realized and we may only increase the costs of manufacturing and care. However, at the local level healthcare providers should consider the impact of putting any product on the skin of their patients. Although the purpose of skin antisepsis is to reduce the burden of organisms on the skin in hopes to reduce the likelihood of infection, it is possible that use of certain products may actually contaminate the skin with organisms capable of causing infection. This is of utmost importance in the critically ill and immunocompromised, those with chronic lung disease such as cystic fibrosis, or those with wounds and indwelling medical devices. Because of these issues, providers should use these products in a targeted fashion when they are labeled as non-sterile and include their use in all-encompassing antimicrobial stewardship programs. Whenever possible, providers should use products labeled as sterile for patients at risk of infection due to organisms with a historical precedence for contaminating the product under use. With everything in healthcare, use of therapies is a risk-benefit calculation.

## Data Availability

Not Applicable. This debate did not include analysis of data; therefore, no data or materials are available.
